# Age-associated changes in microglia activation and Sirtuin-1- chromatin binding patterns

**DOI:** 10.18632/aging.204329

**Published:** 2022-10-10

**Authors:** Liana V. Basova, Nikki Bortell, Bruno Conti, Howard S. Fox, Richard Milner, Maria Cecilia Garibaldi Marcondes

**Affiliations:** 1 San Diego Biomedical Research Institute, San Diego, CA 92121, USA; 2 University of Nebraska Medical Center, Omaha, NE 68198, USA; 3 Oncovalent Therapeutics, Los Angeles, CA 91320, USA

**Keywords:** aging, brain, rhesus macaques, microglia, Sirtuin-1

## Abstract

The aging process is associated with changes in mechanisms maintaining physiology, influenced by genetics and lifestyle, and impacting late life quality and longevity. Brain health is critical in healthy aging. Sirtuin 1 (Sirt1), a histone deacetylase with silencing properties, is one of the molecular determinants experimentally linked to health and longevity. We compared brain pathogenesis and Sirt1-chromatin binding dynamics in brain pre-frontal cortex from 2 groups of elder rhesus macaques, divided by age of necropsy: shorter-lived animals (18-20 years old (yo)), equivalent to 60-70 human yo; and longer-lived animals (23-29 yo), corresponding to 80-100 human yo and modeling successful aging. These were compared with young adult brains (4-7 yo). Our findings indicated drastic differences in the microglia marker Iba1, along with factors influencing Sirt1 levels and activity, such as CD38 (an enzyme limiting NAD that controls Sirt1 activity) and mir142 (a microRNA targeting Sirt1 transcription) between the elder groups. Iba1 was lower in shorter-lived animals than in the other groups, while CD38 was higher in both aging groups compared to young. mir142 and Sirt1 levels were inversely correlated in longer-lived brains (>23yo), but not in shorter-lived brains (18-20 yo). We also found that Sirt1 binding showed signs of better efficiency in longer-lived animals compared to shorter-lived ones, in genes associated with nuclear activity and senescence. Overall, differences in neuroinflammation and Sirt1 interactions with chromatin distinguished shorter- and longer-lived animals, suggesting the importance of preserving microglia and Sirt1 functional efficiency for longevity.

## INTRODUCTION

The aging process is associated with changes in a number of mechanisms maintaining physiology, subjected to the influence of genetics and life style, and leading to a range of outcomes that impact the quality of late life and longevity [1]. Brain health is a critical aspect of healthy aging. Neurological disorders have for several years remained as a leading cause of disability and the second leading cause of death globally [2], and an intrinsic problem of aging [2–4]. Normal aging includes reduction in the efficiency of DNA repair, inflammation, and changes in processes affecting neuronal circuitry [1]. Studies in animal models have suggested the beneficial contribution of genes that modulate lifespan by means that allow survival in conditions of energy availability [5]. In addition to the right genetic variants, acquired epigenetic control may also play a critical role. One of the genes that has been linked to longevity and successful aging, daf16, also known as FOXO, is responsive to the insulin growth factor 1 (IGF1) [6], but also to Sirtuin-1 (Sirt1) [7, 8], an epigenetic regulator, both regarded as molecular determinants of healthy aging.

Sirt1 has gained attention as a type III deacetylase acting on proteins and chromatin histones, to regulate molecular functions and to silence gene transcription in the presence of nicotinamide dinucleotide (NAD+) [9–13]. Sirt1 deacetylates histones H3, H4 and H1 and more than 50 non-histone proteins, including transcription factors and DNA repair proteins [14]. Sirt1 properties contribute to preventing disease by reverting cellular senescence, maintaining genomic integrity and promoting longevity. Increased Sirt1 expression promotes survival in a mouse model of genomic instability and suppresses age-dependent transcriptional changes [15], including of inflammatory genes. In the brain, Sirt1 levels and function have shown to be compromised in neurodegenerative conditions, particularly the ones associated with aging [16, 17]. Sirt1 is also a key factor in blood brain barrier (BBB) integrity and permeability, both directly in microvascular endothelium and indirectly via microglia [18–20]. In infections of the Central Nervous System (CNS), including with Simian Immunodeficiency Virus (SIV) [21], which is a model of Human Immunodeficiency Virus (HIV) [22–25] triggering cellular senescence markers, a drastic decrease in Sirt1 levels and changes in its activity are detectable in isolated microglia cells [21, 26]. The changes in Sirt1 dynamics identified in SIV infection were similar to what was observed in the brain of uninfected macaques with advanced age [26]. In spite of the evidence of Sirt1 as a factor in successful aging, a comparative analysis in subgroups of aged subjects, with animals that differ in health, inflammation, and longevity, has never been previously performed. Sirt1 has been suggested as one of the mediators of the benefits of calorie restriction to longevity [27], associated to decreased intracellular nicotinamide (NAM) [28, 29] and increased levels of nicotinamidases that regenerate NAD+ levels [30], linked to energy metabolism [31]. Sirt1 deficits on the other hand, show increased inflammation, cellular stress, cancer, disrupted glucose and fatty acid metabolism, and unhealthy aging phenotypes [32].

Here, we have compared brain pathogenesis and Sirt1 dynamic chromatin binding differences in brain pre-frontal cortex (PFC) from elder macaques, divided in 2 groups based on the age of necropsy and health conditions. One group consisted of shorter-lived elder animals between 18 and 20 years old (yo), equivalent to 60-70 human yo. Another group consisted of longer-lived elder animals between 23 and 29 yo, corresponding to 80-100 human yo and modeling successful aging. These groups were also compared with young adult 4-7 yo macaques’ brains. We compared neuroinflammatory markers and factors that interfere with Sirt1 levels and activity in the prefrontal cortex (PFC), which is a critical area controlling cognitive functions, including sustained and selective attention, inhibitory control, working memory, and multitasking abilities, which are all impacted by aging [33–35]. Microglia and inflammatory markers included Iba1, CD163 and also CD38, an enzyme that regulates its cellular NAD substrate with consequences to Sirt1 functional activation [36, 37]. Blood brain barrier integrity was accessed by fibrinogen. We also measured transcription of mir142-5p, a micro RNA that targets Sirt1 gene transcription [21]. Sirt1 chromatin binding patterns were compared in total PFC between the two groups of aged rhesus macaques. This allowed the identification of networks of genes and biological processes that may influence longevity in a Sirt1-dependent manner.

## MATERIALS AND METHODS

### Monkeys

SIV-negative, simian retrovirus type D-negative, and herpes B virus-free rhesus macaques with 4-7 years old, purchased from Valley Biosystems (West Sacramento, CA, USA) as controls to other studies, were included in the comparison of molecular and pathological findings across the lifespan. At necropsy, the young animals were terminally anesthetized, and perfused intracardially with sterile PBS containing 1 U/mL of heparin, prior to brain harvest. Young brain frontal cortex samples were frozen and formalin-fixed for histology and used in this study. The brains from 8 elder macaques with ages between 18 and 29 years old were kindly donated by the NIH National Institute of Aging Non-Human Primate Tissue Repository, at the Wisconsin National Primate Center, which is a source of archived tissue from aged nonhuman primates, collected under approved protocols. Upon tissue request, animals that were found dead were excluded to prevent issues with tissue quality. Frozen tissue and paraffin embedded pre-frontal cortex (PFC) sections were made available from animals subjected to necropsy following veterinary recommendations, euthanized using Beuthanasia D (Intervet/Merck Animal Health), under Wisconsin National Primate Center guidelines that are available in primatedatabase.org. The experiments performed in at the San Diego Biomedical Research Institute using primate brain tissues were exempt from Institutional Animal Care and Use Committee, on grounds of repurposing specimens from other approved protocols, and approved by the Institutional Review Board and Biosafety Committees at SDBRI, with Biological Hazard Registration (BHR #20-001-MCM), following National Institutes of Health guidelines.

The characteristics of the animals can be visualized in Table 1.

**Table 1:** Animals used in this study, ages and group assignments.

**Number**	**Age of death (yo)**	**Post-mortem observations**	**Weight (kg)**	**Group assignment**
516	6.74 (scheduled)	Normal	9.6	Young
332	5.48 (scheduled)	Normal	8.5	Young
382	5.55 (scheduled)	Normal	8.3	Young
357	4 (scheduled)	Normal	N/A	Young
39	20.5	Weight loss, diarrhea, dehydration, locally invasive ileocecal carcinoma	7.65	Shorter-lived (18-20yo)
51	19	Weight loss, diarrhea, small intestine membrane carcinoma, secondary amyloidosis in the gut	15	Shorter-lived (18-20yo)
66	18.4	Weight loss, dehydration, enlarged liver, amyloid deposits in liver, spleen, kidney and adrenals	7.4	Shorter-lived (18-20yo)
81	18.25	Weight loss, dehydration, diabetes mellitus, pancreatic amyloidosis, HVP2+, STLV1+	15.1	Shorter-lived (18-20yo)
208	26.75	Weight loss, liver amyloidosis, adhesions, scoliosis of the spine	8.3	Longer-lived (23-29yo)
213	23.42	Weight loss, diarrhea, adhesions, HBV+, STLV1+	7.62	Longer-lived (23-29yo)
227	23.1	Scrotal hernia with resection and anastomosis, small leakage to jejunum, liver cysts, spondylosis and vertebrae fusion, left accessory lung lobe pale purple	11.8	Longer-lived (23-29yo)
325	29.2	Weight loss, dermatitis, ankylosis of joints, kyphosis, tartar and arthritis, liver amyloidosis, renal cortical cyst, constriction of ceco-colonic junction, metaplastic focus on pancreas	8.31	Longer-lived (23-29yo)

N/A – Not available.

### qRT-PCR

RNA and miRNA were extracted from 0.5cm^3^ PFC tissue fragments, using RNeasy and miRNeasy kits, respectively. The qPCR primers for mir-142-5p, mir-142-3p and mir-34a were from the Qiagen miScript Primer Assay using U6 small nuclear RNA (snRNA) as housekeeping control. For qRT-PCR, the RT2 SYBR green qPCR master mix was used with Sirt1 primers and GAPDH was used as housekeeping control. All reagents were Qiagen.

### ChIP-Seq

ChIP was performed in ~0.6cm^3^ tissue fragments by Active Motif (Carlsbad, CA, USA). A ChIP reaction was carried out with 32ug of chromatin (pooled 8ug from each animal per group in duplicate) and anti-Sirt-1 antibody (Millipore). The ChIP DNA was processed into an Illumina ChIP-Seq library and sequenced +/10000 bb, to generate >2 million reads, which were aligned to the M.mulatta genome annotation (MacaM/December 2019 assembly) and >15 million unique aligns (removed duplicates) were obtained. A signal map showing fragment densities along the genome was visualized in the Integrated Genome Browser (IGB) and MACS peak finding was used to identify peaks. Control data was derived from 5.1 million (positive control) and 5.8 million (negative control) alignments. With default settings, 307 Sirt-1 meaningful peaks genome-wide consistent to promoter regions in all samples, were identified. Raw data and metadata are available at GEO GSE95793.

### Systems analysis

Pathway assignments and functional annotations were analyzed using DAVID Bioinformatics Database [38]. To complete the bioinformatics analysis, two knowledge base resources were queried: the Ingenuity Knowledge Base [39] and interaction repositories based on cpath [40–42] containing interactions that have been curated by GeneGo (http://www.genego.com), the Kyoto Encyclopedia of Genes and Genomes (KEGG - http://www.genome.jp/kegg/), and Ingenuity. Benjamini False Discovery Rate (FDR) adjusted values <0.01 and p values < 0.05 (provided by DAVID) were utilized as conservative filters for identification of true values. Cluster analysis and networks were obtained and visualized using Cytoscape 3.9.1 [43]. Pathway and genetic interaction-based connections between significantly different genes were assembled and visualized using GeneMania. Active pathways were identified using JActive Modules based on score and low (0.8) overlap threshold.

### Immunohistochemistry (IHC)

Formalin-fixed, paraffin embedded brain tissue sectioned in 7u slices was used for the detection of molecular markers using antibodies against Iba-1 (AIF1 - WAKO, Richmond, VA, USA), CD163 (Invitrogen), Fibrinogen (Millipore, Temecula, CA, USA) and CD38 (Novus Biologicals, Centenial, CO, USA), using standard procedures [44]. The incubation with biotinylated secondary antibodies (Vector Labs, Burlingame, CA, USA) was followed by Streptavidin-HRP, and development was performed using NovaRed (Vector Labs), and counterstaining with Gill’s hematoxylin.

### Statistical analysis

Results are expressed as Mean ± SD. One-way analysis of variance with Bonferroni post hoc test and Student’s *t* test were performed in Prism 8 (GraphPad Software LLC). Pearson analysis, graph builder properties and full factorial analysis were performed in JMP Pro15. *P* < 0.05 was considered significant.

## RESULTS

### Inflammation markers differentiate between young, shorter- and longer-lived elder animals

**Figure 1:**
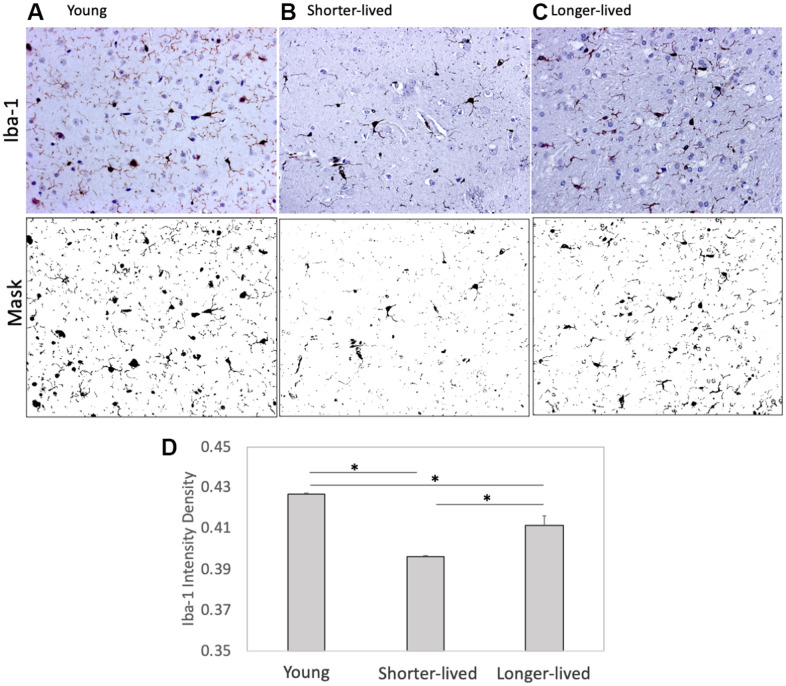
**Expression of the microglia marker Iba1 in PFC from young animals and animals that lived to age.** Paraffin-embedded tissue sections were stained with antibody anti- Iba-1 (AIF1, dark brown staining) and digitally imaged for density intensity quantification in whole section digital 8-bit images and binary masks using Image J (NIH). (**A**) Representative image taken from a young (4-7 yo) monkey, (**B**) representative shorter-lived (18-20 yo) group image, from an 18 yo monkey (M66), (**C**) representative longer-lived (23 – 29 yo) group image, from a 27 yo monkey (M208). (**D**) Iba-1 intensity density measured in ImageJ Fiji, using mask features. N=4/group. All representative images are 20X magnification. *p<0.0001 in multiple comparisons.

Paraffin-embedded tissue from animals in Table 1 was used for identifying differences in the expression of microglia markers using IHC (Figure 1). Iba-1 (AIF1), and CD38 were measured and quantified using Image J. We found that PFC from elder animals, regardless of group assignment, differed significantly from young PFC (Figure 1). However, these differences occurred in different ways within the aged group, in an age-dependent fashion. For instance, shorter-lived elder animals, which died between 18 and 20 years of age, showed a significantly smaller number of Iba1+ cells compared to young ones, and to longer-lived animals, indicating that severe microglial loss was a characteristic of the group with shorter lifespan (F2,12=189.2, p<0.0001). Microglia morphology and Iba1 quantification in longer-lived elder animals indicated some impact of age, with significant although less severe microglial loss compared to shorter-lived animals. The results of Iba1+ cell morphology and density suggested that the ability to maintain the microglia population may be important for longevity.

**Figure 2:**
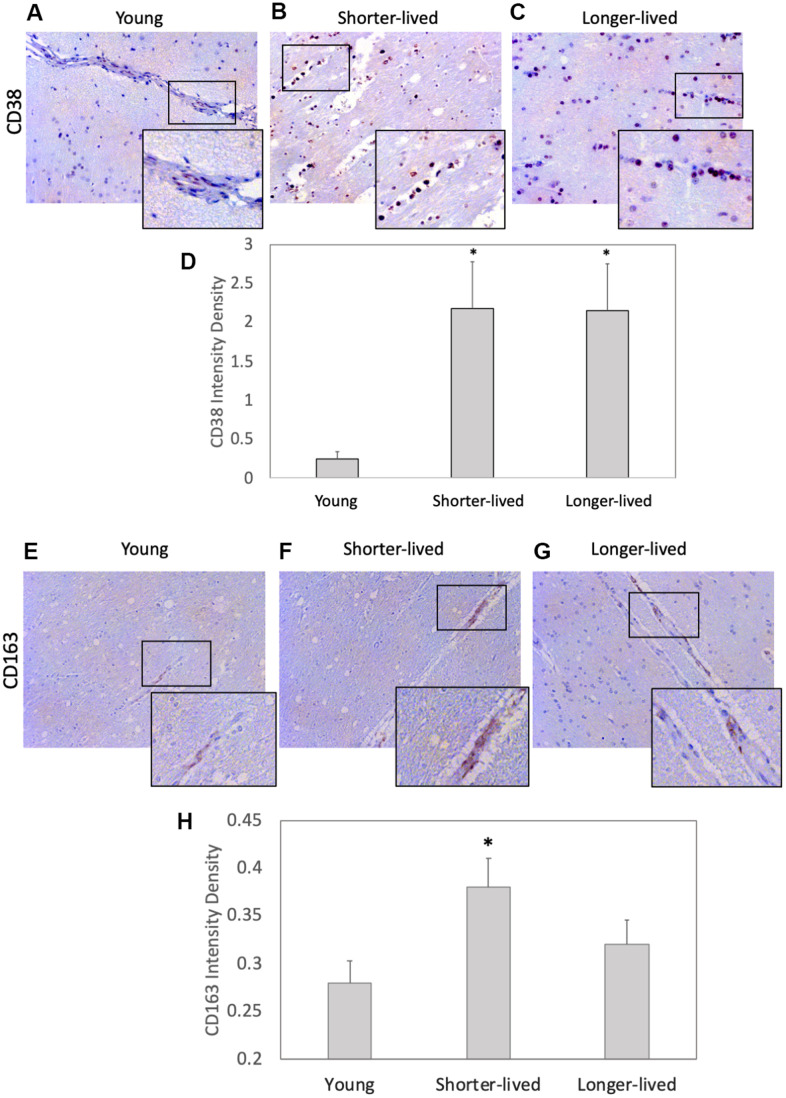
**Expression of the inflammation markers CD38 and CD163 in PFC from young and elder animals.** Paraffin-embedded PFC sections were stained with (**A**–**C**) antibody anti- CD38 (observed in brown color) and (**E**–**G**) anti- CD163, which were (**D**, **H**) digitally imaged for density intensity quantification in 8-bit binary masks using Image J (NIH). Rectangles indicate areas expanded for detail. (**A**) Representative CD38 image of young (4-7 yo) group, (**B**) representative CD38 image of shorter-lived elder animals. (**C**) Representative CD38 image of longer-lived elders. (**D**) CD38 staining density intensity measured in 8-bit digital whole section images and binary masks, using ImageJ. (**E**) Representative CD163 image of young (4-7 yo) group, (**F**) representative CD163 image of shorter-lived elder animals. (**G**) Representative CD163 image of longer-lived elders. (**H**) CD163 staining density intensity measured in 8-bit digital whole section images and binary masks, using ImageJ. All images are 40X magnification. N=4/group. *p<0.05 in multiple comparisons.

CD38 is a marker of immune activation and a NAD limiting factor. We found an effect of age on the expression of CD38 measured by IHC on PFC sections (F2,12=4.995, p=0.0347). This marker expressed at higher levels in all elder animals compared to young controls (Figure 2D). Young animals had few CD38+ cells associated with vessels and few diffuse in the parenchyma (Figure 2A). Although the two elder groups expressed similar CD38 intensity levels (p=0.998), which were higher than young (p=0.05), distribution patterns differed significantly between them (Figure 2B–2D). In shorter-lived elders, CD38+ cells were strongly stained and were mostly clustered in perivascular foci, associated with signs of edema and tissue damage. On the other hand, in longer-lived animals these cells were diffuse, some perivascular, but no severe pathology. CD163 expression in myeloid cells characterizes response to inflammation and was restricted to the perivascular domain (Figure 2E–2G). Although blood vessels were enlarged in both elder groups, shorter-lived animals had significantly more detectable CD38+ cells compared to young, while in longer-lived animals the increase was not significantly different from the other groups (Figures 2H).

**Figure 3:**
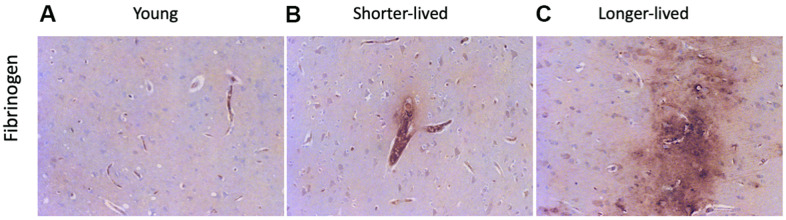
**BBB integrity via detection of fibrinogen in PFC from young and elder animals.** Paraffin-embedded PFC sections were stained with antibody anti- fibrinogen. (**A**) representative image from young (4-7 yo) group, (**B**) representative image from the shorter-lived group, (**C**) representative image from longer-lived animals. All images are 20X magnification.

The loss of integrity of the BBB is a critical component of aging, which can be detectable in tissue sections by the staining against fibrinogen, which is normally maintained within blood vessels by strong endothelial junctions [45]. Fibrinogen staining marked the microvasculature and, when found in the extravascular space, it indicated loss of BBB integrity and leaks (Figure 3). In both elder groups, extravascular fibrinogen was occasionally detected, showing leaks from blood vessels to the brain tissue (Figure 3B, 3C). Larger leaks were observed in the longer-lived group (Figure 3C), compared to the shorter-lived group (Figure 3B).

### Mir-142-5p increased with age while Sirt1 transcription decreased in PFC of long-lived aged animals

**Figure 4:**
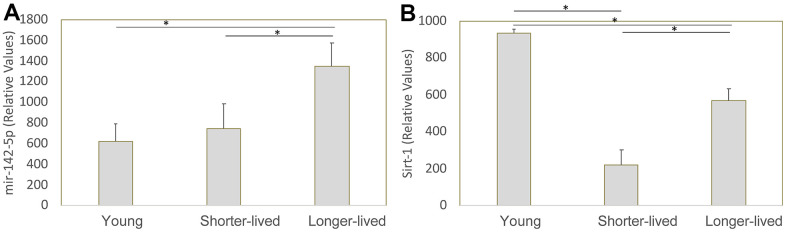
**Transcription of mir-142-5p and Sirt1 genes in PFC from young animals and animals that lived to age.** The expression of these genes was measured by qRT-PCR in total PFC extracted mRNA and normalized against GAPDH. Relative values of (**A**) mir142-5p and (**B**) Sirt1 were compared between young (4-7 yo), shorter-lived (18-20yo) and longer-lived (23 – 29 yo) elder groups. *p<0.05 in Bonferroni’s multiple comparisons.

We previously identified mir142 as a critical contributor to the collapse of Sirt1 transcription and function in macaques that develop neuropathology as a result of infection with the Simian Immunodeficiency Virus [21]. Given the role of Sirt1 in aging, we examined the transcription of mir-142-5p, mir-142-3p and Sirt1 genes in mRNA extracted from the PFC of all young and long-lived aged animals, which were made available to us by the NIA Non-Human Primate Tissue Repository (n=4/group). Mir-142-3p was not detectable (data not shown). Regarding mir-142-5p, shorter-lived elder animals did not differ in its transcription compared to young animals, while longer-lived ones showed significantly higher transcription compared to both young and shorter-lived elders (Figure 4A). Sirt1 transcription was decreased in both elder groups compared to young, however shorter-lived elders had a significantly lower Sirt1 expression compared to longer-lived animals (Figure 4B), indicating a correlation between longevity and maintenance of Sirt1 transcriptional activity.

### Signature changes in Sirtuin -1 binding to chromatin distinguish shorter- and longer-lived elder groups

In order to estimate the link between longevity, Sirt1 transcription and chromatin binding activity, we examined differences in Sirt1 dynamics and binding to chromatin in both groups of elder animals. The comparison and analysis of Sirt1 binding patterns and target genes was indicative of epigenetic silencing activity and signatures associated with longevity and maintenance of microglia cells, in spite of inflammation and vascular leaks.

**Figure 5:**
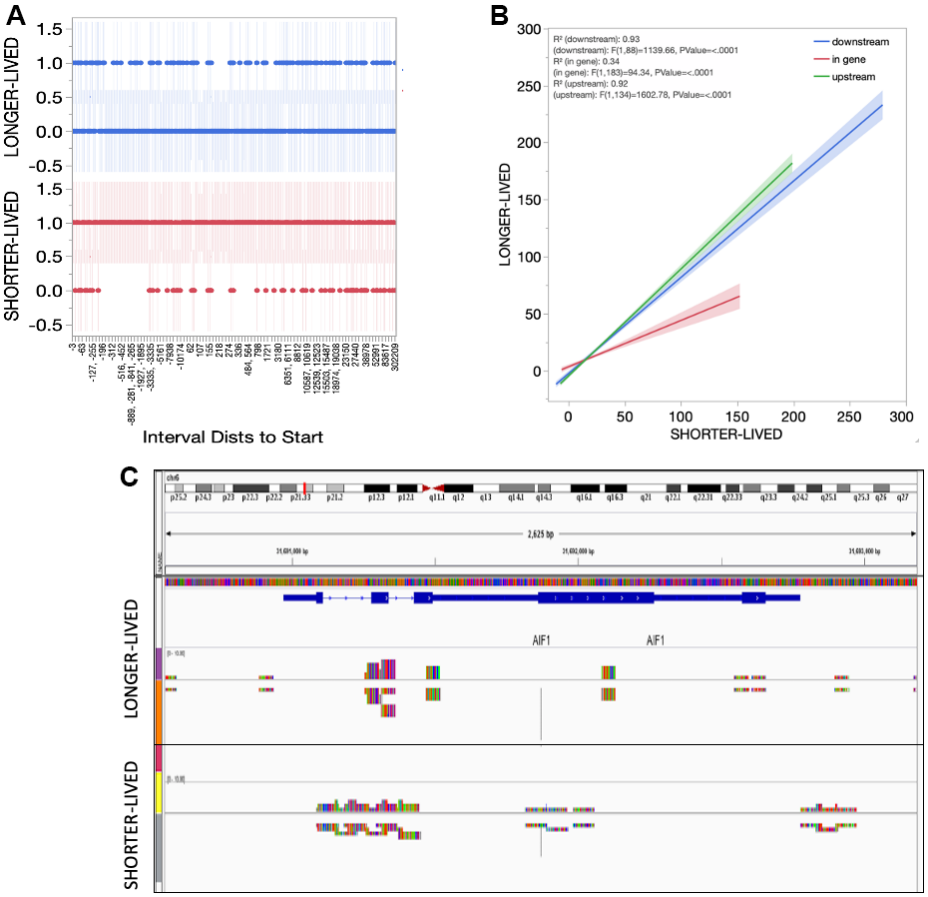
**Sirt1 distribution in the PFC tissue from shorter- and longer- lived elder animals.** (**A**) Interval distances from start where Sirt1 peaks are observed in chromatin preparations from PFC bulk tissue indicating presence (1) versus absence (0). (**B**) Interval positions of Sirt1 binding in PFC from shorter- and longer-lived animals. (**C**) Example of Sirt1 peak signal in the AIF1 gene, indicating a spread in shorter animals.

In spite of higher transcription of Sirt1, the absolute number of Sirt1 peaks was reduced in long-lived aged (>23yo) animals compared to old (18-20) (Table 2). However, the distribution of peaks indicated a smaller diversity in the genes regulated by Sirt1 in long-lived animals compared to old, characterized by gaps in the genomic intervals presenting Sirt1 peak reads in long-lived animals (Figure 5A). On the other hand, the detailed analysis of intervals also indicated and enrichment of in-gene binding sites in old animals compared to long-lived (Figure 5B). The implications of these differences are unknown but may reflect more frequent disruptions in the transcriptional process occurring in old PFC, in addition to the active silencing at regulatory regions. Figure 5C shows an example of these functional amendments in the AIF1 gene that encodes Iba1, indicating a concentration of Sirt1 peak signal on promoter and regulatory regions (resembling controls [26]) in long-lived animals, and a spread of Sirt1 signal in the same gene in old PFC.

**Table 2:** Characterization of Sirt1 peak signal and interval distribution in PFC from shorter- (18-20yo) and longer-lived (>23 yo) elder rhesus macaques.

**Description**	**Act regions**	**Shorter- Sirt1 Rh**	**Shorter- Sirt1 Rh (%)**	**Longer- Sirt1 Rh**	**Longer- Sirt1 Rh (%)**	**Input overlap**	**Input overlap (%)**
Total # Intervals in Build		307		110		34	
# Intervals within -10000/+10000 bp of Genes	274	226	73.62%	75	68.18%	12	35.29%
# Intervals NOT within -10000/+10000 bp of Genes	137	81	26.38%	35	31.82%	22	64.71%
# NCBI Genes with Intervals within -10000/+10000 bp	411	347		107		10	
# Intervals within Promoter Region (-7500/+2500 bp of NCBI Gene Start)	208	181	58.96%	52	47.27%	6	17.65%
# Intervals 500 bp of NCBI Tx Start	145	129	42.02%	37	33.64%	1	2.94%
# NCBI Genes with intervals in Promoters (-7500/2500 bp of start)	304	267		69		10	

**Figure 6:**
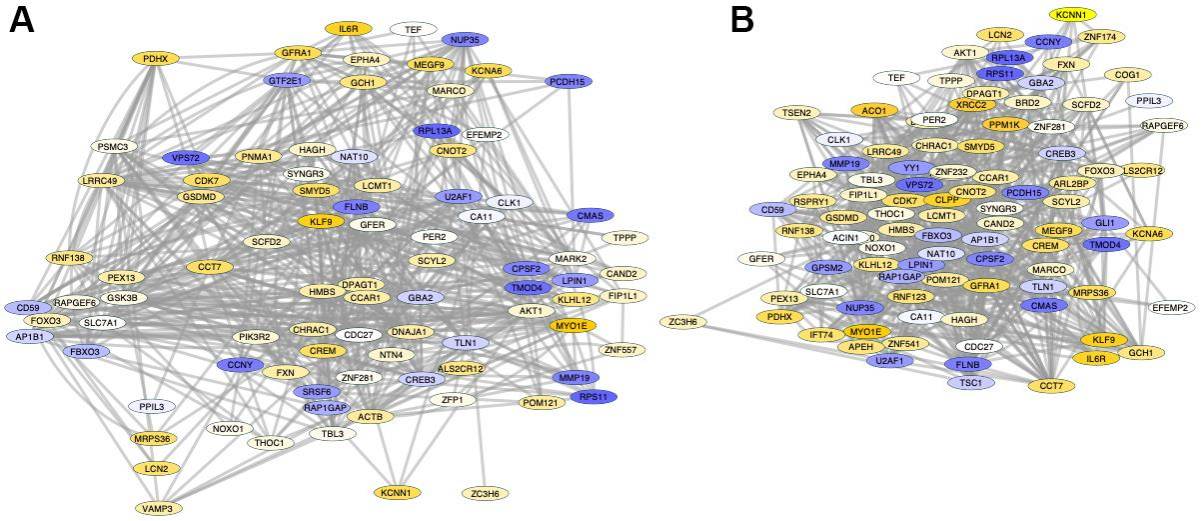
**Pathway-based gene networks with differences in Sirt1 binding in PFC chromatin between shorter- and longer-lived elder animals.** Significant differences in Sirt1 bind peak binding and intensity were introduced in GeneMania for fold-change visualization regardless of genomic region between pooled shorter-lived versus longer-lived elder PFCs. A network of interacting genes consistent in all animals was subjected to JActiveModules for the identification of pathway based modular clusters with minimal overlap. Two pathway -based modular clusters were identified: (**A**) Module 1 (detail in Supplementary Material Module 1 and Table 3). (**B**) Module 2 (detail in Supplementary Material Module 2 and Table 4). Tones of yellow indicate increase, and tones of blue indicate decrease in Sirt1 peaks in shorter-lived animals compared to longer-lived ones, indicating differences in silencing between the groups.

A detailed analysis of changes in Sirt1 binding between PFCs from shorter- and from longer-lived elder animals, regardless of position, indicated differences in the activity of Sirt1 on genes strongly associated by pathway (Figure 6), visualized as fold change (old/long-lived) in Genemania and analyzed using JActive Modules for the identification of active pathway connections between gene clusters with low overlapping threshold. Two main modular networks were identified (Figure 6). Module A connected 90 genes with a score 5.9 (Figure 6A). Module B connected 99 genes with a score 5.52 (Figure 6B). The enrichment in Sirt1 activity in these networks may signify processes that are actively disrupted or downregulated in shorter-lived animals compared to longed-lived elders, and vice-versa.

Module A (Figure 6A) contained 47 out of 90 genes that interacted through pathway which exhibited Sirt1 binding activity significantly increased above 1.5-fold in shorter- compared to longer-lived, indicating that they are more likely to being silenced in shorter-lived animals but active in longer-lived ones. These genes were annotated to transferase molecular functions (p=0.0063), mitochondrion as a cellular component (p=0.0068), and several biological processes, including transport (p=0.0011), heme biosynthesis (p=0.0033(p=0.0046) and protein transport (p=0.0052). KEGG pathway assessments indicated that these genes were involved in EGFR tyrosine kinase inhibitor resistance, endometrial cancer, longevity regulation, prolactin signaling, HIF-1 signaling, Neurotrophin signaling, Thyroid hormone signaling, FoxO signaling, cellular senescence, and the JAK/STAT pathway (Table 3). In the same module A, 17 genes had a -1.5 decrease in Sirt1 binding in shorter- versus longer-lived animals, indicating that these genes are more active in shorter-lived animals, but being more tightly regulated in longer-lived PFCs. These were annotated to pathways such as RNA binding molecular functions (p=0.0035), nucleus as a cellular component (p=0.039), and mRNA processing biological process (p=0.0058).

**Table 3:** KEGG pathway annotations for module 1.

**Pathways**	**P-Value**
EGFR tyrosine kinase inhibitor resistance	0.003
Focal adhesions	0.009
Endometrial cancer	0.015
Longevity regulating pathway	0.017
GnRH secretion	0.018
Prolactin signaling pathway	0.021
Non-small cell lung cancer	0.022
Neutrophil extracellular trap formation	0.022
Shigellosis	0.043
HIF-1 signaling pathway	0.047
Neurotrophin signaling pathway	0.047
AMPK signaling pathway	0.048
Thyroid hormone signaling pathway	0.049
FoxO signaling pathway	0.05
Cellular senescence	0.051
JAK-STAT signaling pathway	0.052

Module B contained 57 out of 99 genes interacting through pathway (Table 4) which exhibited Sirt1 binding activity increased above 1.5-fold, indicating that they may be silenced in shorter-lived PFCs but active in longer-lived ones. Mitochondrion was annotated as a cellular component (p=0.011). Biological processes associated with these genes (Table 4) were protein kinase activity (p=0.009), heme biosynthesis (p=0.04) and iron transport (p=0.049). The EGFR tyrosine kinase biosynthesis was the only pathway annotation identified for these genes (p=0.02). In module B, 19 genes had a -1.5 decrease in Sirt1 binding, indicating that they were active in shorter-lived animals but being likely regulated in longer-lived PFC. Similar to module A, these were annotated to nucleus as a cellular component (p=0.008), with differentiation as a biological process (p=0.0052).

**Table 4:** KEGG pathway annotations for module 2.

**Pathways**	**P-Value**
Longevity regulating pathway	0.002
Cytoplasmic transport	0.003
AMPK signaling pathway	0.004
EGFR tyrosine kinase inhibitor resistance	0.008

The genes with highest Sirt1 peaks in shorter-lived PFCs compared to longer-lived ones (most likely silenced) included Myosin 1E (MYO1E, 4-fold), Kruppel-like factor 9 (KLF9, 3.75-fold), Forkhead box O3 (FOXO3, 2.86-fold) and Interleukin 6 Receptor (IL6R, 3.6-fold). Genes with highest Sirt1 peaks in longer-lived animals compared to shorter-lived (indicating likely suppression in longer-lived) included the ribosomal protein 13a (RPL13A, 0.13-fold), the ribosomal protein S11 (RPS11, 0.13-fold), the matrix metallopeptidase 19 (MMP19, 0.19-fold), Tropomodulin 4 (TMOD4, 0.19-fold), N-acylneuraminate cytidylyltransferase (CMAS, 0.23-fold) and Cyclin Y (CCNY, 0.25-fold), associated with RNA binding (p=0.011) and acetylation processes (p=0.053), all regulated by Sirt1.

Of interest to the regulation of the longevity process, FOXO3 (2.86-fold) and AKT serine/threonine kinase (AKT1, 1.9-fold) had Sirt1 peaks in shorter- versus longer-lived animals. The TSC complex subunit 1 (TSC1) and cAMP responsive element binding protein 3 (CREBP3) had significantly less (0.7-fold) Sirt1 binding in shorter- versus longer-lived PFCs.

## DISCUSSION

Differences in the Iba1 microglia compartment between shorter – and longer-lived PFC and compared to young indicated that microglia loss may be a component affecting longevity. Shorter-lived animals not only had significantly less microglial cells than longer-lived and young, but also showed more signs of tissue damage with edema and perivascular CD163+ cells, as well as lower transcription of Sirt1. Interestingly, both elder groups had similar levels of CD38+ cells, which was higher than young, and BBB leaks.

Microglia activation and BBB integrity both contribute to small vessel disease that is commonly found in aging, although not necessarily in an interdependent manner [46]. However, microglial activation has been observed around vascular leaks, including in models that replicate the aging brain such as mild hypoxia [47]. Importantly, microglial depletion drastically increases loss of tight junction proteins that characterize vascular integrity, largely aggravating leaks [48]. Microglia cells may interact with extravascular fibrinogen to promote protective signals [48], balancing pro-inflammatory responses. Paradoxically, occasional but larger leaks were observed in longer-lived animals, which had microglial cell at levels that were higher than shorter-lived ones. This observation could support a role for fibrinogen and microglia signal interactions on maintaining the homeostasis of brain cell populations and BBB integrity. Whether microglial cell numbers and Sirt1 transcription and activity determine protection and survival outcomes, remains to be addressed.

The CD38+ cells, previously linked to neurodegenerative and neuroinflammatory insults of aging [49], were present in both elderly groups at levels that were higher than in young PFC. Yet, their distribution differed considerably, being associated to perivascular edema and tissue damage in shorter-lived animals, but diffuse and lightly stained in longer-lived animals. CD38 can be expressed by both T cells and macrophages, playing a critical role in pro-inflammatory responses strongly linked to its enzymatic activity over NAD, and as a prognosis of pathogenic outcome [50, 51]. Importantly, NAD serves as a neuroprotective agent [36] by activating Sirtuins activity [52].

The regulation of Sirt1 and its gene silencing functions, can result from both transcriptional changes, as well as changes impacting its functional dynamics. We have previously described the role of mir-142 in controlling Sirt1 transcription in microglia [21]. However, the results indicate that mir-142 may be one of the factors controlling Sirt1 transcription. For instance, longer-lived animals had higher levels of mir-142, yet Sirt1 transcription was lowest in shorter-lived animals. Other microRNAs have been suggested to regulate Sirt1, such as mir-34a [53], which occurred at low levels in these specimens and did not differ between groups (data not shown). Sirt1 transcription could be influenced by transcription factors not addressed in this study, such as E2F1 and HIC1, particularly in conditions of oxidative stress [54, 55].

The comparison between elderly groups of Sirt1 chromatin binding sites and frequency in bulk PFC tissue by ChIP-seq served as an indirect measure of epigenetic function and silencing activity. Chromatin binding peaks differed significantly in quality, as well as in numbers, between the two elderly groups indicating divergent patterns and regulated processes. The comparison between PFC of shorter-lived animals, young (4-7yo) controls, as well as young rhesus macaques infected with SIV, has been previously described by us [26]. That comparison indicated that a decrease in Sirt1 activity in conditions of SIV infection was similar to age [26]. The comparison of aging subgroups performed here indicated qualitative differences associated with longevity outcomes in uninfected animals. Interestingly, shorter- and longer-lived animals showed Sirt1 peak differences in genes associated with aging, metabolism and nuclear activity. Sirt1 binding to in-gene sequences was significantly enriched in shorter-lived elder animals, with no changes in upstream and downstream activity was similar between the elder groups. The implications of in-gene silencing are not well defined. It is possible that this may serve as a mechanism of transcriptional disruption, generating truncated or non-functional RNA, in addition to the active silencing in promoters and regulatory intron regions, and resulting in transcript degradation [56]. Whether this is a factor that results from or contributes to the lack of microglia cells, remains to be addressed.

Although Sirt1 activity was concentrated in genes involved in aging pathways (HIF, senescence, longevity, etc.), interesting differences were detected in individual genes between the two elder groups. Shorter-lived animals, for example, had strong Sirt1 enrichment in the IL6R gene, indicating its silencing. This could be a consequence of lower microglia cell numbers, or defective inflammatory responses. Enrichment patterns indicate that longer-lived animals have Sirt1 activity in RNA binding and acetylation genes, but lower activity in nuclear and differentiation pathways.

Microglia are the first cells to populate the brain during development [57, 58], preceding neurogenesis and the formation of BBB [59, 60]. RNAseq studies have suggested that subsets of microglia become more prevalent with age, which may be associated with protection [61].

This study has limitations due to the small number of animals. Moreover, the differences between elderly groups could be linked to clinical observations leading to necropsy and to post-mortem findings. For instance, long-lived animals had compromised bones and joints, and internal adhesions, but did not have tumors, diabetes or signs of metabolic disorders, which were rather found in the shorter-lived elderly group. Thus, microglial loss could also result from systemic disease more frequently observed in shorter-lived animals. Age-matching controls, particularly to the shorter-lived animals’ group was not available, limiting conclusions. Yet, it is unlikely that the loss of microglia is just a transient age-effect, while the maintenance of microglia numbers in animals that lived longer suggests this may be a hallmark of healthy aging. The correlation between microglia and age was striking, suggesting that supporting this population may be critical to longevity.

Successful aging is largely associated with preserved cognition [62], for which data is not available in the animals studied here. This work suggests that preservation of microglia and Sirt1 binding activity and patterns, as well as the directionality of pathways regulated by Sirt1, are prognostic of long-living. This work suggests the importance of microglia and epigenetic cross-talk in aging processes, influenced by and influencing pathogenesis.

## Supplementary Material

Supplementary Material Module 1

Supplementary Material Module 2
